# Screening of Parkinson's Differential MicroRNA Based on GEO Database and Its Clinical Verification

**DOI:** 10.1155/2021/8171236

**Published:** 2021-11-13

**Authors:** Xuping Jiang, Lili Xiao, Xumei Jiang, Guangsheng Li, Zhijuan Lu

**Affiliations:** ^1^Department of Neurology, Ganzhou People's Hospital, Ganzhou 341000, Jiangxi Province, China; ^2^Department of Pharmacy, Ganzhou Fifth People's Hospital, Ganzhou 341000, Jiangxi Province, China

## Abstract

**Objective:**

This study is set out to explore the potential difference of miR in PD through GEO data and provide diagnostic indicators for clinical practice.

**Methods:**

In this study, differential miR was screened through the Gene Expression Omnibus (GEO) database, 68 PD patients treated in our hospital from May 2017 to March 2018 were collected as the research group (RG), and 50 normal subjects who underwent physical examination in our hospital during the same period were collected as the control group (CG). Quantitative real-time polymerase chain reaction (qRT-PCR) was used to detect the expression and diagnostic value of miR-374a-5p in serum of patients. The potential target genes of miR-374a-5p were predicted, and Kyoto Encyclopedia of Genes and Genomes (KEGG) analysis and Gene Ontology Consortium (GO) were carried out.

**Results:**

GEO2R analysis revealed that 193 miRs are expressed differentially, of which 78 were highly expressed and 115 were poorly expressed. The miR-374a-5p expression in the serum of the RG was reduced markedly and had a diagnostic value. Targetscan and miRDB online websites were used to predict their target genes, with 415 common target genes. miR-374a-5p may participate in 27 functional pathways and 8 signal pathways.

**Conclusion:**

miR-335-5p has low expression in PD and is expected to be a potential diagnostic indicator.

## 1. Introduction

Parkinson's disease (PD) is the second clinically common neurodegenerative disease after Alzheimer's disease, characterized by gradual loss of dopaminergic neurons in the substantia nigra [1, 2]. Statistics reveal that [3, 4] patients over 60 years old have one PD patient per 800-1000 people, and the incidence of that line is higher than that of women. At present, the relevant mechanism of PD is not clear. Studies have found that [3] PD may be caused by deletion of SNCA and PINK1 genes. However, this disease is sporadic, and its pathogenesis involves environment, epigenetic, etc. In addition, it has a long latent period, the early stage of the disease has no great influence on patients, and the lack of medical knowledge leads to their admission to enter the middle and late stages [5, 6]. With the continuous improvement of the medical level in recent years, PD prevention has been popularized, but patients' daily life will be seriously affected in the middle and late stages of the disease [7].

MicroRNA (miR) is a short-chain noncoding RNA with a length of 20-25 nucleotides [8]. Studies show that [9, 10] miR can regulate downstream target genes by regulating the 3′ untranslated region of downstream target gene mRNA. Previous studies have found that miR is involved in the occurrence and development of various diseases such as tumors, cardiovascular diseases, and neurological diseases [11–13]. Some studies have revealed that there are differential expression levels of various miRs in PD. For example, some studies have found that the miR-221 expression in the serum of [14] PD patients reduces, which is expected to become a potential diagnostic indicator of PD. Other studies have found that [15] miR-155 can regulate the inflammatory response of the Parkinson's disease model induced by *α*-synuclein. The GEO database is one of the largest public chip databases in the world [16], which contains gene chips for various diseases. We have found that there are many differences in miR through analysis of the chips in the database this time, but it has not been clinically verified.

Therefore, in this research, we explored the differential genes in expression profile dataset GSE16658 and carried out clinical verification to find potential observation indicators for clinical use.

## 2. Materials and Methods

### 2.1. Patient Data

Sixty-eight PD patients treated in our hospital from May 2017 to March 2018 were collected as the research group (RG), and another 50 normal patients who underwent physical examination in our hospital during the same period were collected as the control group (CG). There was no statistical difference between the two groups in age and gender. This study was approved by the Medical Ethics Committee of our hospital. Inclusion criteria include patients who met Parkinson's diagnostic criteria and those who met Hoehn and Yahr (H&Y) staging criteria [17]. Patients and their families were informed, and they all signed an informed consent form. Exclusion criteria include patients who were secondary or iatrogenic PD, who passed the mini-mental state examination (MMSE) score, and who had renal insufficiency, multiple tumors, and stroke.

MMSE refers to the test that measures global cognitive functioning on domains that include memory, attention, language, praxis, and visuospatial ability. The summed scores of MMSE ranged from 0 to 30, with higher values denoting better cognitive function.

### 2.2. GEO Chip Analysis

We checked Parkinson's disease microRNA-related chips by logging into the Gene Expression Omnibus (GEO) database (https://www.ncbi.nlm.nih.gov/gds) and selected GSE16658 for analysis. The miRNA expression dataset GSE16658 was downloaded from the National Center for Biotechnology Information GEO database (http://www.ncbi.nlm.nih.gov/geo). In the GSE16658 dataset, expression profiles were obtained from peripheral blood mononuclear cells from patients with PD and normal controls and were quantified using the miRCURY LNA microRNA Array, v.10.0-hsa, mmu & rno (Exiqon A/S, Vedbæk, Denmark). The analysis was performed by GEO2R software built in the GEO database. miRs with the top 10 differences were selected for heat mapping. The chip data are shown in Table 1.

### 2.3. Collection of Test Samples

We collected 5 mL of peripheral venous blood from people in the two groups and then subpacked it to the blood collection tube (5 mL) of inert separation gel and coagulant. Then, it was centrifuged at 3000 rpm for 10 min at 24°C to collect part of serum for subsequent experiments, and the surplus part was placed in an EP tube without RNA enzyme for later use at -80°C.

### 2.4. Quantitative Real-Time Polymerase Chain Reaction (qRT-PCR) Detection

Total RNA was extracted from the collected serum by the TRIzol kit (Invitrogen Company, USA), and its purity, concentration, and integrity were detected by ultraviolet spectrophotometer and agarose gel electrophoresis. Subsequently, reverse transcription was carried out using the TaqMan™ reverse transcription kit (Invitrogen Company, USA), and the transcription steps were strictly operated according to the kit instructions. The obtained cDNA was subjected to subsequent research. PCR amplification was carried out using the PrimeScript RT Master Mix kit (Takarabo Company, Japan). The amplification system was as below: 10 *μ*L SYBR qPCR Mix, 0.8 *μ*L for upstream and downstream primers, 2 *μ*L cDNA product, 0.4 *μ*L 50x ROX reference dye, and RNase-free water supplemented to 20 *μ*L. PCR reaction conditions were as below: 95°C predenaturation for 60 s, 95°C denaturation for 30 s, and 60°C annealing extension for 40 s, with a total of 40 cycles. In the experiment, three parallel repeating holes were designed, and all specimens were repeatedly tested 3 times. miR used U6 as the internal reference and used 2^-*ΔΔ*ct^ to analyze the data [18]. The PCR instrument was 7500PCR instrument from ABI Company.

### 2.5. Bioinformatics Analysis

The online target gene prediction website was used to predict the target gene, and then, the clusterProfiler package in R software was used to carry out Kyoto Encyclopedia of Genes and Genomes (KEGG) analysis and Gene Ontology Consortium (GO) and draw pictures.

### 2.6. Statistical Analysis

In this study, the SPSS20.0 software package was used to carry out statistical analysis on the collected data. GraphPad Prism 7 was used to draw the data picture. The usage (%) of the counting data was confirmed by the chi-squared test and expressed by *χ*^2^. The K-S test was employed to analyze the data distribution. The measurement data were expressed by the mean ± standard deviation (means ± SD). The comparison of normal distribution data between the two groups was conducted by the independent-samples *t*-test, which was expressed by *t*. ROC was used to analyze the diagnostic value of miR-374a-5p in PD. A *p* value lower than 0.05 was statistically different.

## 3. Results

### 3.1. Analysis of Chip Results

**Figure 1:**
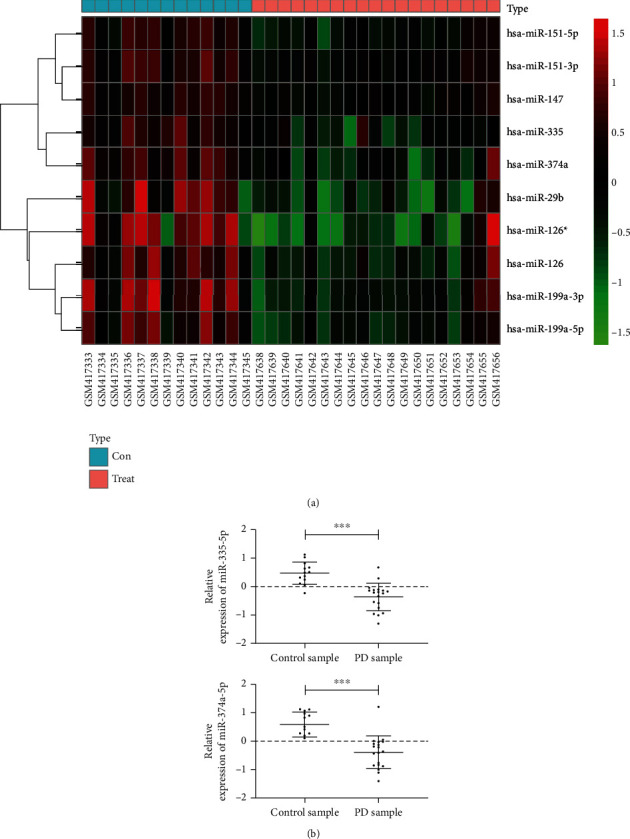
miR with top 10 differences in GSE16658 chip. (a) miR with significant differences in the top 10. (b) Expression of miR-335-5p and miR-374a-5p. ∗∗∗ indicates *p* < 0.001. A *p* value lower than 0.001 was statistically different.

Through analysis by online analysis software GEO2R, we found 193 miRs with differences, including 78 with high expression and 115 with low expression. We selected the top 10 miRs for display and combined the references and adjusted differences. Finally, we selected miR-335-5p and miR-374a-5p for further research. More details are shown in Figure 1 and Table 2.

### 3.2. Expression of miR-335-5p and miR-374a-5p in PD Patients

**Figure 2:**
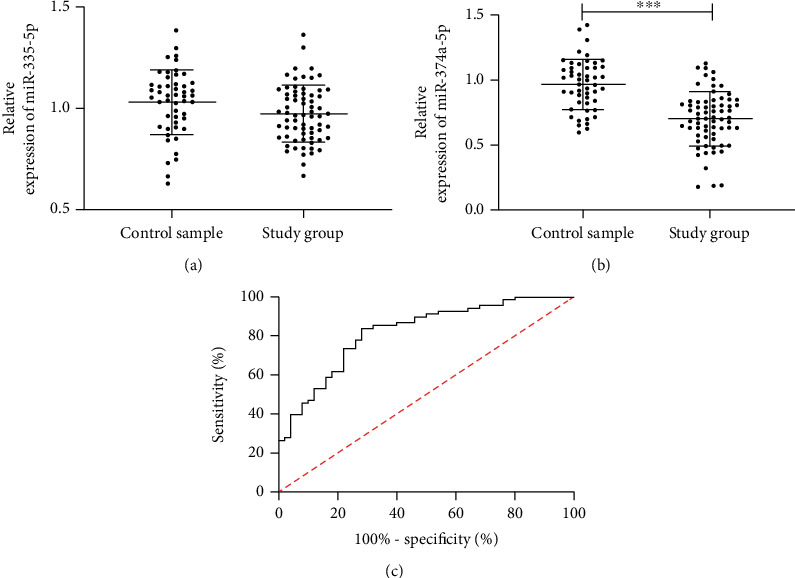
Expression and diagnostic value of miR-335-5p and miR-374a-5p in PD. (a) Expression of miR-335-5p in PD patients. (b) Expression of miR-374a-5p in PD patients. (c) ROC curve of miR-374a-5p in diagnosis of PD. ∗∗∗ indicates *p* < 0.001. A *p* value lower than 0.001 was statistically different.

We detected the miR-335 and miR-374a-5p expression in the serum of patients. The results revealed that the miR-335-5p expression in the serum of the RG was not different from that in the CG, while the miR-374a-5p expression in the serum of patients reduced remarkably. Furthermore, we found that miR-374a-5p had a certain clinical value in the diagnosis of PD by drawing ROC curve analysis, and the area under the curve was 0.820. More details are shown in Figure 2.

### 3.3. Relationship between miR-374a-5p and H&Y Stage

**Figure 3:**
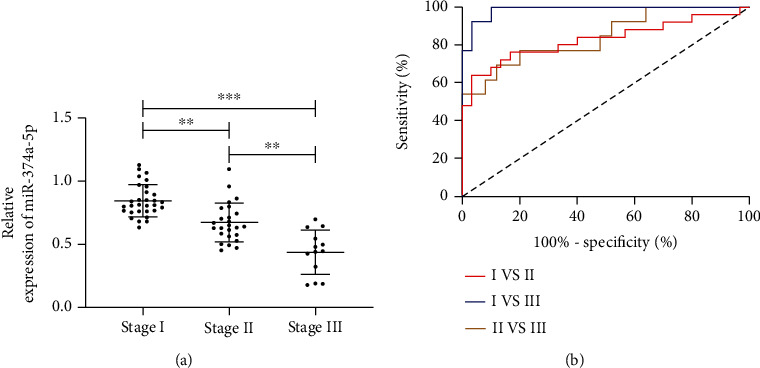
Relationship between miR-374a-5p and H&Y stage. (a) Expression of miR-374a-5p in different stages. (b) Diagnostic value of miR-374a-5p in distinguishing different stages.

We divided patients into three stages according to the H&Y stage, namely, the first stage (H&Y stage: 1-2, *n* = 30), the second stage (H&Y stage: 3-4, *n* = 25), and the third stage (H&Y stage: 5, *n* = 13). We further detected the miR-374a-5p expression in patients of different stages and explored that miR-374a-5p was differentially expressed in the three stages. The ROC curve analysis displayed that miR-374a-5p had high clinical value in distinguishing patients at different stages. More details are shown in Figure 3 and Table 3.

### 3.4. Bioinformatics Analysis

**Figure 4:**
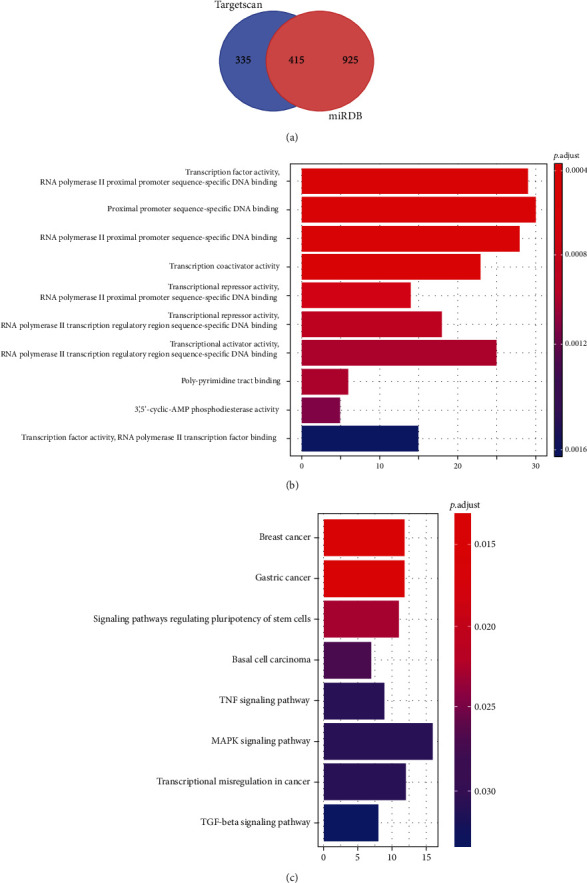
Prediction and functional analysis of miR-374a-5p target gene. (a) miR-374a-5p target genes were predicted jointly by Targetscan and miRDB. (b) Top 10 functional pathways for GO enrichment. (c) Signal pathway for KEGG enrichment.

In order to further explore the relevant mechanism of miR-374a-5p, we predicted its target genes through the online websites of Targetscan and miRDB. The results demonstrated that 415 common target genes existed. To further determine its potential mechanism, we employed the R software clusterProfiler package for GO and KEGG enrichment analysis. The results exhibited that miR-374a-5p might participate in 27 functional pathways and 8 signal pathways. More details are shown in Figure 4 and Tables 4 and 5.

## 4. Discussion

PD is a serious nervous system disease. The gradual loss of dopaminergic neurons in substantia nigra is one of the primary causes of PD [19]. What is more, due to the long incubation period of PD, patients are not clear about its early symptoms, which leads to PD patients being in the middle and late stages, thus causing them to miss the best treatment period [20]. Therefore, we urgently need to explore the relevant mechanism of PD.

In recent years, more and more studies have found a close relationship between miR and PD. For instance, Ma and others [21] pointed out that serum miR-221 could be used as a biomarker of Parkinson's disease. Other studies have discovered that [22] inhibition of miR-34b and miR-34c enhances the *α*-synuclein expression in Parkinson's disease, thus improving the disease condition. All the above studies signify that miR is involved in the occurrence and development of PD. In order to better screen potential PD differences of miR, we analyzed based on PD chips in the GEO database and found significant differences between miR-335-5p and miR-374a-5p, and the differences between the two after correction were the same, so we chose them for research.

Previous studies on miR-335-5p, miR-374a-5p, and PD are very few. To verify the expression and value of the two miRs in PD, we tested them. As a result, we found that there was no difference in the miR-335-5p expression in the serum of PD patients, while the miR-374a-5p slashed. Moreover, we also plotted the ROC curve and verified that miR-374a-5p had a high clinical value in distinguishing PD from normal people. Previous studies have found that miR-374a-5p is expressed in breast cancer, esophageal cancer, colon cancer, and other tumors and participates in the occurrence and development of tumors through relevant mechanisms [23–25]. This study was the first time we found that miR-374a-5p was differentially expressed in PD. To further determine the clinical value of miR-374a-5p in PD, we also analyzed the diagnostic value of miR-374a-5p in different H&Y stages. The H&Y stage is an important clinical score used to distinguish PD, which is divided into 5 stages. In this study, we further divided the patients into 3 stages based on the H&Y stage. Previously, Zhang and others divided them into groups according to this scheme. After grouping patients, we further detected the miR-374a-5p expression in patients' serum. At last, we confirmed that miR-374a-5p was differentially expressed in those at different stages through Figure 3(a), and its expression decreased with the increase of stages, which indicated that PD was tied to the miR-374a-5p expression in the serum of severe patients. In addition, through ROC curve analysis, we found that miR-335-5p had a high clinical value in distinguishing patients of different stages, and the area under the curve was more than 0.8. Through the above research, we preliminarily confirmed the clinical value of miR-335-5p in PD, but we are still unclear about its relevant mechanism.

To further determine the relevant mechanism of miR-335-5p, we predicted its downstream target genes. Through the joint prediction of two online websites, we found 415 potential target genes downstream of miR-335-5p. Further enrichment analysis discovered that miR-335-5p participated in many functional pathways and signal pathways. Among them, the TNF signaling pathway, MAPK signaling pathway, and TGF-beta signaling pathway are important signal pathways for PD occurrence [26–28]. For instance, previous studies have found that [29] TNF signal inhibition in the central nervous system has an impact on normal brain function and neurodegenerative diseases. Other studies have verified that [30] activation of the MAPK signal pathway aggravates the condition of PD patients. Besides, studies have found that [31] TGF-*β* plays a role in the development, maintenance, and neuroprotection of dopamine neurons. Nevertheless, the analysis of this life letter provides us with the direction for future research.

There are still some limitations in this study. First of all, we only collected patient serum samples, and the samples were single. Some studies have found that differential expression of miR has also been detected in cerebrospinal fluid and peripheral blood mononuclear cells (PBMC). Moreover, the number of samples is relatively small, which may affect the data. Last but not least, we have not conducted in-depth research on the mechanism of miR-335-5p. Although we have conducted the credibility analysis to provide ideas for our research, we have not conducted experiments to confirm it. Hence, we hope to add more experiments and samples in future research to improve our research results.

To summarize, miR-335-5p has low expression in PD and is expected to become a potential diagnostic indicator.

## Figures and Tables

**Table 1:** Baseline data.

Data	GSE16658
Time	
Submission date	Jun 17, 2009
Last update date	Jan 13, 2016
Contact name	Sofia A Oliveira
Address	
Organization name	Instituto de Medicina Molecular
Department	Edificio Egas Moniz
Street address	Av. Prof Egas Moniz
City	Lisbon
Country	Portugal
ZIP/postal code	1649-028
Organism	Homo sapiens
Experiment type	Noncoding RNA profiling by array
Platforms	GPL7722, miRCURY LNA microRNA Array, v.10.0-hsa, mmu & rno

**Table 2:** Top 10 differences of miR.

miRNA	Adjusted *p* value	*p* value	*t* value	*β*	log FC
miR-335	1.730*E* − 03	8.720*E* − 06	-5.261	3.540	-0.831
miR-374a	1.730*E* − 03	9.140*E* − 06	-5.245	3.498	-0.978
miR-199a-3p	1.750*E* − 03	1.650*E* − 05	-5.044	2.961	-1.126
miR-126^∗^	1.750*E* − 03	2.234*E* − 05	-4.941	2.686	-1.593
miR-151-3p	1.750*E* − 03	2.320*E* − 05	-4.928	2.652	-0.614
miR-199a-5p	1.750*E* − 03	2.769*E* − 05	-4.868	2.491	-0.951
miR-151-5p	2.070*E* − 03	4.702*E* − 05	-4.687	2.010	-0.719
miR-126	2.070*E* − 03	4.713*E* − 05	-4.686	2.008	-0.985
miR-29b	2.070*E* − 03	4.915*E* − 05	-4.672	1.969	-1.236
miR-147	2.130*E* − 03	6.340*E* − 05	-4.584	1.738	-0.496

Note: log FC: fold change.

**Table 3:** ROC parameters.

Factor	AUC	95% CI	Specificity	Sensitivity	Youden index	Cut-off
I vs. II	0.828	0.709-0.947	96.67%	64.00%	60.67%	0.680
I vs. III	0.987	0.963-1.000	90.00%	100.00%	90.00%	0.700
II vs. III	0.843	0.705-0.981	88.00%	69.23%	57.23%	0.498

**Table 4:** Top 10 terms for GO enrichment.

ID	Description	*p* value	Gene ID	Count
GO:0000982	Transcription factor activity, RNA polymerase II proximal promoter sequence-specific DNA binding	2.872*E* − 05	MEF2D, MECP2, HES1, DACH1, EN1, NFIL3, ZBTB20, NR3C1, PITX2, RORB, ZNF281, HOXA10, NEUROD1, NR4A3, BCL11B, GABPA, GATA3, KLF8, SP1, MYT1L, ATF2, MSX1, BHLHE40, SP3, FOSB, RFX4, ASCL1, CEBPB, ONECUT2	29
GO:0000987	Proximal promoter sequence-specific DNA binding	2.872*E* − 05	MEF2D, HES1, RFX3, EN1, NFIL3, NR3C1, PITX2, RORB, ZNF281, ZNF516, HOXA10, NEUROD1, NR4A3, BCL11B, GABPA, GATA3, CHD7, KLF8, SP1, ATF2, NEUROG2, BHLHE40, SP3, FOSB, RFX4, ASCL1, NKX2-2, CEBPB, ONECUT2, SMAD6	30
GO:0000978	RNA polymerase II proximal promoter sequence-specific DNA binding	1.024*E* − 04	MEF2D, HES1, RFX3, EN1, NFIL3, NR3C1, PITX2, RORB, ZNF281, HOXA10, NEUROD1, NR4A3, BCL11B, GABPA, GATA3, CHD7, KLF8, SP1, ATF2, NEUROG2, BHLHE40, SP3, FOSB, RFX4, ASCL1, CEBPB, ONECUT2, SMAD6	28
GO:0003713	Transcription coactivator activity	1.024*E* − 04	ACTN4, TAF5L, TAF4B, RNF14, HCFC1, PPARGC1A, PITX2, RORB, NEUROD1, KAT6A, SP4, GABPA, GATA3, RORA, TFDP1, JMY, ATF2, TCERG1, MED13, NCOA1, MED12L, ACTN1, NKX2-2	23
GO:0001078	Transcriptional repressor activity, RNA polymerase II proximal promoter sequence-specific DNA binding	3.497*E* − 04	MECP2, HES1, DACH1, EN1, NFIL3, ZBTB20, ZNF281, GATA3, KLF8, MYT1L, BHLHE40, SP3, ASCL1, CEBPB	14
GO:0001227	Transcriptional repressor activity, RNA polymerase II transcription regulatory region sequence-specific DNA binding	4.575*E* − 04	MECP2, HES1, DACH1, EN1, FOXD3, NFIL3, ZBTB20, ZNF281, MLX, GATA3, KLF8, ZC3H8, MYT1L, MSX1, BHLHE40, SP3, ASCL1, CEBPB	18
GO:0001228	Transcriptional activator activity, RNA polymerase II transcription regulatory region sequence-specific DNA binding	5.664*E* − 04	MEF2D, HOXA1, HCFC1, NR3C1, PITX2, RORB, HOXA10, PKNOX1, NEUROD1, NR4A3, BCL11B, GBX2, GABPA, FOXD2, GATA3, RORA, TFDP1, SP1, ATF2, MSX1, ETV5, FOSB, RFX4, CEBPB, ONECUT2	25
GO:0008187	Poly-pyrimidine tract binding	5.664*E* − 04	PNPT1, UHMK1, IFIT5, MSI2, ATXN1, PABPC1	6
GO:0004115	3′,5′-Cyclic-AMP phosphodiesterase activity	7.791*E* − 04	PDE4D, PDE3A, PDE7B, PDE10A, PDE8B	5
GO:0001076	Transcription factor activity, RNA polymerase II transcription factor binding	1.373*E* − 03	NR3C1, PPARGC1A, PITX2, RORB, NEUROD1, GBX2, RORA, ATF2, TCERG1, MED13, NCOA1, BHLHE40, ARHGAP5, CDC73, MED12L	15

**Table 5:** KEGG enrichment terms.

ID	Description	*p* value	Gene ID	Count
hsa05224	Breast cancer	0.013	HES1, WNT3, WNT5A, WNT16, GADD45A, SP1, FZD5, FGF18, NCOA1, FGF5, AKT1, APC	12
hsa05226	Gastric cancer	0.013	WNT3, FGFR2, WNT5A, WNT16, GADD45A, FZD5, FGF18, CCNE2, FGF5, AKT1, APC, HGF	12
hsa04550	Signaling pathways regulating pluripotency of stem cells	0.020	WNT3, FGFR2, WNT5A, KAT6A, INHBB, WNT16, FZD5, ACVR2B, AKT1, SMARCAD1, APC	11
hsa05217	Basal cell carcinoma	0.025	WNT3, WNT5A, BMP2, WNT16, GADD45A, FZD5, APC	7
hsa04668	TNF signaling pathway	0.028	MAP2K6, VEGFC, MAP2K4, CCL2, MMP14, ATF2, AKT1, CEBPB, BIRC3	9
hsa04010	MAPK signaling pathway	0.028	MAP2K6, VEGFC, FGFR2, MAP2K4, DUSP6, GADD45A, MAP3K2, RASA2, ATF2, FGF18, STK4, NTF3, TGFA, FGF5, AKT1, HGF	16
hsa05202	Transcriptional misregulation in cancer	0.028	AFF1, HOXA10, NR4A3, BCL11B, WNT16, DUSP6, GADD45A, SP1, ETV5, HOXA11, CEBPB, BIRC3	12
hsa04350	TGF-beta signaling pathway	0.031	PITX2, BMP2, INHBB, TFDP1, SP1, NEO1, ACVR2B, SMAD6	8

## Data Availability

The data used during the present study are available from the corresponding author upon reasonable request.
